# Retraction: Cesium cation additive in PbI_2_ layer regulates crystal growth and carrier dynamics in air processed MAPbI_3_ perovskites solar cells

**DOI:** 10.1039/d6ra90164f

**Published:** 2026-10-30

**Authors:** Annisa Zahra Ahdaliza, Nurul Iffah Ismail, Ikhwan Fikri Maulidan, Gufran Umar Alam Shaikh, Muhammad Aniq Shazni Mohammad Hanif, Suhaila Sepeai, Puvaneswaran Chelvanathan, Norasikin Ahmad Ludin, Akrajas Ali Umar

**Affiliations:** a Institute of Microengineering and Nanoelectronics (IMEN), Universiti Kebangsaan Malaysia Bangi 43600 Malaysia akrajas@ukm.edu.my; b Solar Energy Research Institute (SERI), Universiti Kebangsaan Malaysia Bangi 43600 Malaysia

## Abstract

Retraction of ‘Cesium cation additive in PbI_2_ layer regulates crystal growth and carrier dynamics in air processed MAPbI_3_ perovskites solar cells’ by Annisa Zahra Ahdaliza *et al.*, *RSC Adv.*, 2026, **16**, 42604–42621, https://doi.org/10.1039/d6ra03921a.

We, the named authors, hereby wholly retract this *RSC Advances* article due to the inclusion of confidential data.

The underlying work was conducted with external funding support that included an industry partner. During preparation and publication of the manuscript certain data were included that should have remained confidential as they were intended to be withheld in support of a subsequent patent process and filing. The inclusion of those data in the published article was an inadvertent oversight by the authors. The authors recognize the seriousness of that oversight and, upon identifying its implications, wish to address it transparently and without delay.

The authors are therefore retracting this article in consideration of intellectual-property concerns.

Signed: Akrajas Ali Umar (on behalf of all authors)

Date: 23rd October 2026

Retraction endorsed by Laura Fisher, Executive Editor, *RSC Advances*

